# Prevalence and Species Identifications of Camel Ixodid Ticks in Habru District, North Wollo Zone, Northeast Ethiopia

**DOI:** 10.1002/vms3.70338

**Published:** 2025-04-08

**Authors:** Tsedalu Yirsa, Yidersal Tizazu, Abebe Berihun, Asres Zegeye

**Affiliations:** ^1^ Department of Veterinary Medicine College of Agriculture Woldia University Woldia Ethiopia; ^2^ Sirinka Agriculture Research Center Woldia Ethiopia

**Keywords:** camel, Habru, identification, prevalence, ticks

## Abstract

**Background:**

Ticks are among the most prevalent ectoparasites and carriers of important infectious diseases in animals and people worldwide, particularly in tropical and subtropical regions.

**Methods:**

A cross‐sectional study from December 2023 to April 2024 sought to determine the incidence and species of camel ixodid ticks in the Habru district. Ticks were categorized into genera and species using morphological identification under a stereomicroscope.

**Findings:**

A total of 1008 ixodid ticks were collected from 384 randomly selected camels and identified to species level. The overall tick infestation rate was 55.21% in the study camels. Poor body condition and lack of deworming were significantly associated with higher mature tick infestations (*p value* <0.05). Nevertheless, there were no significant variations in prevalence among animals of different ages (*p value* >0.05). The most common tick species in this study was *Hyalomma dromedarii* (28.7%), followed by *Amblyomma variegatum* (23.02%) and *Rhipicephalus pulchelis* (21.63%). However, *Amblyomma lepidium* (4.2%) was the least common tick species. Except for *Boophilus decolaratus*, all tick species had male‐predominant sex ratios. The maximum tick infection was identified beneath the animals’ tails (30.06%), whereas the lowest infestation was found on the necks (4.46%). Ticks were commonly found across the study area, and animals were afflicted with various ticks.

**Conclusions:**

Generally, these ticks are well‐known for generating enormous economic losses by transmitting various infectious diseases and disrupting animal health and output. Thus, efficient tick control methods should be adopted in the area.

## Graphical abstract

The overall tick infestation rate was 55.21% in camels. The most common tick species was *H*. *dromedarii* (28.7%). Most tick species had male‐predominant sex ratios. They were more located beneath the animals' tails (30.06%). Ticks were commonly found in the study area.

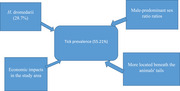

**Abbreviations**
CSA: Central Statistical Authority
TBD: tick born disease

## 1 Introduction

Ethiopia boasts one of the largest camel populations globally, ranking third in Africa after Somalia and Sudan (Elias et al. 2020; Hussen and Agonafir 2018). The country is home to approximately 1.06 million camels, predominantly found in its arid and semi‐arid regions, especially in Borana, Ogaden and Afar provinces (Elias et al. 2020). The one‐humped camel (*Camelus dromedarius*) is a species of significant socioeconomic importance, uniquely adapted to harsh environments and variable nutritional conditions in arid and extreme desert zones (Dinka et al. 2010; Feki et al. 2021; Feyera Dewo et al. 2017). Camels provide essential resources such as milk, meat, hide, drought relief and transportation (Feyera Dewo et al. 2017; Al‐Salihi 2016). They inhabit predominantly dry‐ and semi‐arid regions unsuitable for agricultural cultivation and less favourable for traditional animal husbandry (Dinka et al. 2010). With ongoing land degradation and increasing human populations, camels are expected to play an even more vital role in the future (Bekele 2010). However, camels are highly vulnerable to various diseases and parasitic infestations (Al Salihi 2020). External parasites affecting camels include ticks, mites and other arthropods such as myiasis flies, all of which pose serious threats to their health and productivity (El Tigani and Mohammed 2010). Among these, ticks are the most significant parasite impacting the health, productivity and overall performance of camels (Bekele 2010; Kiros et al. 2014).

Ticks are among the most widespread ectoparasites and critical vectors of animal diseases globally, especially in tropical and subtropical regions (El Tigani and Mohammed 2010). Ixodid ticks, which are obligate hematophagous ectoparasites, are recognized as major carriers of various infections (Ali et al. 2023). In Ethiopia, the primary tick genera include *Amblyomma, Boophilus, Haemaphysalis, Hyalomma* and *Rhipicephalus* (Taddese and Mustefa 2013). Notably, *Rhipicephalus pulchellus, Amblyomma gemma* and *Hyalomma dromedarii* are the most common tick species found on camels in Eastern Ethiopia (Elias et al. 2020; Dinka et al. 2010; Jama et al. 2023; Megersa et al. 2012).

Ticks play a vital role in transmitting infectious diseases, which pose serious global health risks to both humans and animals, with numerous viruses capable of infecting multiple species (Perveen et al. 2021). These ectoparasites are key vectors for a variety of pathogens, including bacteria, protozoa, rickettsiae and viruses, causing conditions such as blood loss, irritation, inflammation, hypersensitivity and skin damage (Kiros et al. 2014; Alanazi et al. 2018; Barghash et al. 2016). Additionally, ticks transmit diseases to animals, including babesiosis, anaplasmosis, heartworm and lyme disease (Jama et al. 2023). Camels can also act as carriers of zoonotic diseases, transmitting infections to humans (El‐Alfy et al. 2024).

Tick‐borne diseases (TBDs) pose the greatest threat to Ethiopia's livestock production systems (Jama et al. 2023). Although past studies have examined the prevalence of tick infestations and species identification in camels across the country (Elias et al. 2020; Hussen and Agonafir 2018; Feki et al. 2021; Feyera Dewo et al. 2017; Kiros et al. 2014; Jama et al. 2023). Ethiopia's diverse environmental and climatic zones have contributed to the proliferation of various tick species (Taddese and Mustefa 2013; Sileshi et al. 2014). However, tick infestations in pastoral regions remain under‐researched due to limited veterinary services and reliance on traditional epidemiological methods (Jama et al. 2023). There is also limited knowledge about tick infestations in the country's highland areas, and no studies have specifically focused on camel ectoparasites in the study area. Consequently, understanding the occurrence and geographic distribution of tick species is crucial for preventing TBDs and infections (Kiros et al. 2014). This study aims to assess the prevalence, identify tick species and evaluate risk factors associated with camel hard ticks in the Habru district.

## 2 Materials and Methods

### 2.1 Study Area and Period

**FIGURE 1:**
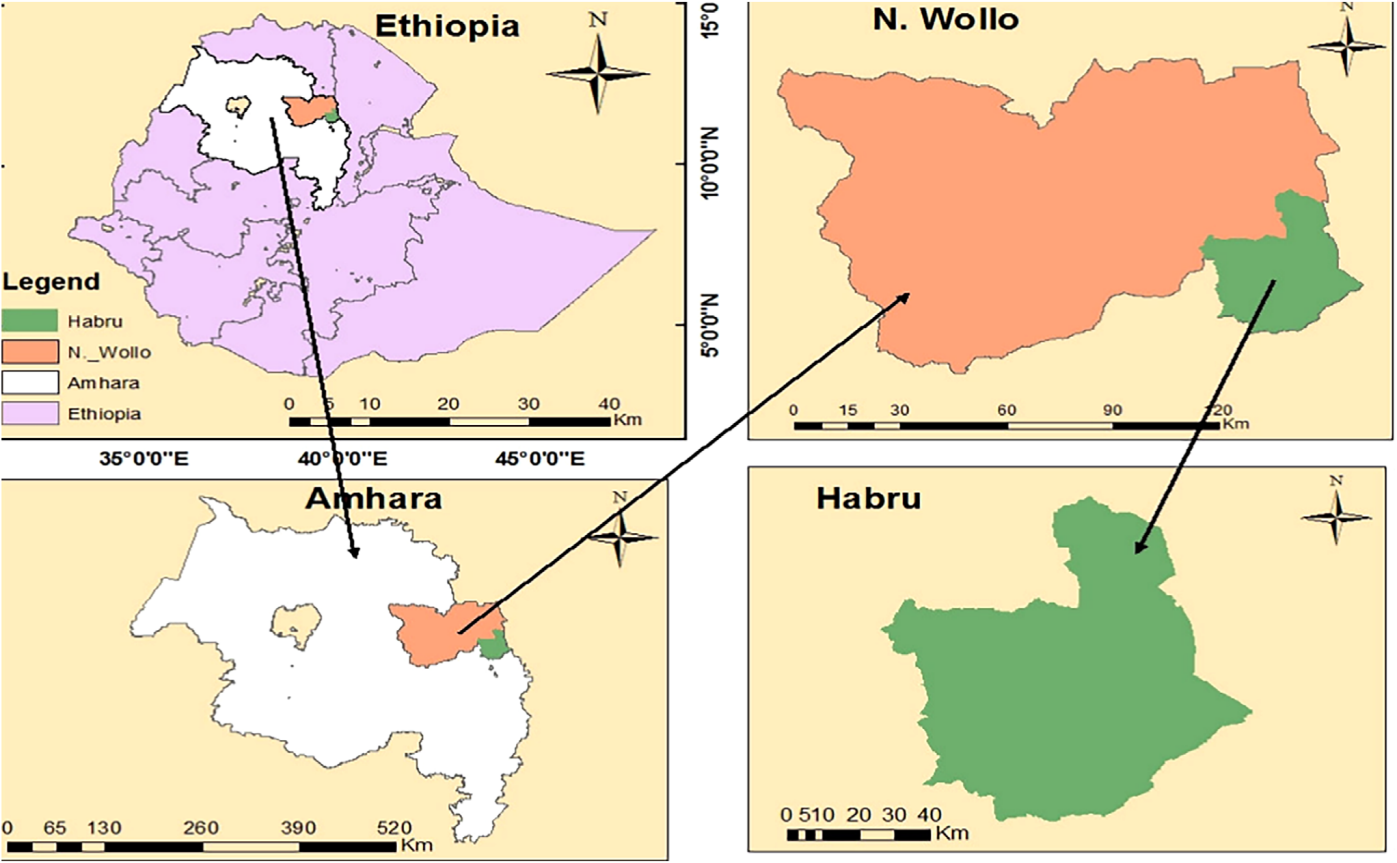
Geographical location map of Habru district. *Source*: GIS map (2017).

This study was conducted in the Habru district, part of the North Wollo Administrative Zone in Ethiopia's Amhara National Regional State, from December 2023 to May 2024. The district is bordered by the Mille River to the south, Gubalafto Woreda to the west, Kobo Woreda to the north and the Afar region to the east (Figure 1). It covers a total area of 1239.79 km^2^ (123,979 ha) and comprises 36 rural kebeles and 3 sub‐urban kebele administrations. Its administrative centre, Mersa, is situated 491 km northeast of Addis Ababa and 30 km south of Woldia, the capital of the North Wollo Zone (OHWARD (Office of Habru Woreda Agriculture and Rural Development) 2016). The districts lie at a latitude of 11°44′59.99″N, a longitude of 39°39′59.99″E and an elevation of 1625 m above sea level. The district's weather is divided into three climate zones: *dega* (temperate) at 3.5%, *kolla* (tropical) at 56.5% and *weinadega* (subtropical) at 40%. It has an average annual temperature of 21.5°C, with monthly ranges from 10.6°C to 30.8°C. it has annual rainfall averages 1045 mm, following a bimodal pattern, with peaks in March–April and July–October, and a dry season from November to February (SARC [Sirinka Agriculture Research Center] 2015). The district has a population of 192,742, primarily residing in rural areas, resulting in a population density of 155.46 persons per square kilometre, higher than the zone average. The dominant religions are Islam (76.85%) and Ethiopian Orthodox Christianity (22.95%) (Alemu et al. 2024). Habru is one of the most drought‐prone and food‐insecure areas in the zone. Economic activities mainly revolve around large commercial farms focused on livestock rearing and agroprocessing, especially in the Girana kebele. The district benefits from its strategic location along the main road connecting it to Addis Ababa, facilitating market access. Local products are sold in nearby markets, including Mersa and Dessie towns.

### 2.2 Study Populations and Putative Variables

The study was conducted on a one‐humped camel (*C. dromedarius*) found in the Habru district. Camels of all ages and body conditions (BCs) were included in this study. The study assessed factors influencing tick distribution, including age, treatment and BC, which are linked to biological difference and previous tick prevalence reports in various parts of Ethiopia (Elias et al. 2020; Jama et al. 2023; Ahmed et al. 2024; Alemu and Abebe 2023). Younger camels have weaker immune systems, making them more susceptible to ticks, whereas older camels may face increasing exposure. Camels in poor BC are also more vulnerable to ticks due to weakened defences, whereas those in good condition are better able to resist infestations (Feki et al. 2021; Jama et al. 2023; Ahmed et al. 2024). Regular tick treatments can reduce tick prevalence compared to untreated animals, with variations in treatment frequency and methods further affecting tick distribution (Jama et al. 2023). The age of camels was divided into young (<2 years); adult (2–4 years) and old (>4 years old following the guidelines of Alemu and Abebe (2023)). The camels’ BC was also classified as poor, medium and good based on the guidelines of Faye et al. (2001). The previous treatments of the camels were whether dewormed or non‐dewormed based on the recorded and surveyed of their owners during sample collection.

### 2.3 Study Design

A cross‐sectional research was undertaken from December 2023 to May 2024 to determine the prevalence, species identification, and associated risk factors of the Camel Ixodid tick in Habru district, North Wollo zone, Northeast Ethiopia.

### 2.4 Sample Size Calculations and Sampling Methods

The sample size was calculated using the formula given by Thrusfield (2007) for the simple random sampling approach. Because no previous research had been conducted in this area, the sample size was determined using 50% predicted prevalence. As a result, the sample size for this investigation was estimated using the following formula:

$$N=\frac{1.96^{2}\left(P_{\exp}\right)\left(1-P_{\exp}\right)}{d^{2}}$$

where *N* is the sample size required; 1.96 is the value of *Z* at 95% confidence interval; *P*_exp_ is the expected prevalence (50%), and *d* is the desired absolute precision (5%). Hence, the required sample size, as determined using the formula mentioned above, was 384 camels. These camels were selected through a simple random sampling method using a lottery system from the camel population in the study area. Ticks were collected from multiple anatomical sites on each camel, yielding a total of 1008 ixodid ticks, which were identified to the species level.

### 2.5 Tick Collection and Identifications Techniques

Camels were meticulously inspected for ticks with the aid of their owners. Tick‐predilection regions on the animals’ bodies, such as the head, neck sternum, under the tail, ventral, scrotum/udder and back/side surface, were meticulously evaluated using eye examination and skin palpation. All apparent adult ticks clinging to these places of the animals’ bodies were carefully and gently removed. The collected ticks were then stored in a properly labelled tick‐collecting vial containing 70% alcohol. The bottles were labelled with the date of collection, location, age and body size and then delivered to the Woldia University Microbiology Laboratory for tick identification.

The collected ticks from each bottle were placed onto Petri dishes and examined under a stereomicroscope to identify the species level using tick identification (Elias et al. 2020; Hussen and Agonafir 2018; Jama et al. 2023). Briefly, the main identification features of the ticks were scutum, anal groove, festoon (ornamentation), Mouthparts and leg colour for genes and species identification of ticks.

### 2.6 Data Management and Analysis

The data were entered, cleaned and coded in MS Excel, then analysed using STATA version 17 software. Descriptive statistics, including proportions, relative prevalence, percentages and ratios, were calculated to assess tick prevalence across variables and tick species distribution across body sites. Univariable logistic regression was used to examine associations between risk factors and tick infestation. Variables with a *p* value ≤0.05 were included in a multivariable logistic regression model. The final model employed multivariable mixed‐effects logistic regression for variables that were statistically significant in the univariable analysis. Odds ratios were used to quantify associations, with a 95% confidence interval to evaluate precision and significance. A 5% absolute precision level determined the significance of differences between parameters.

## 3 Findings

### 3.1 Proportion of Sampled Animals Across the Category of Predictor Variables

**FIGURE 2:**
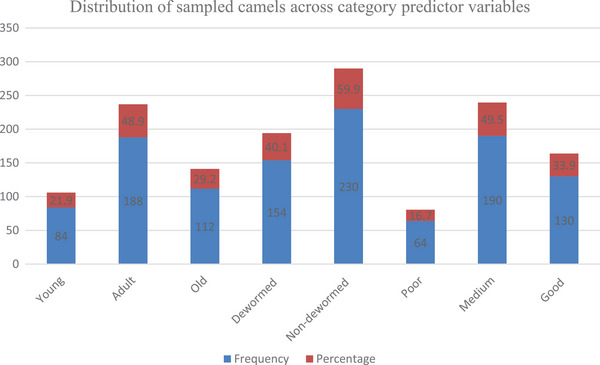
Graphical distribution of sampled camels across variable categories.

Descriptive information about the proportion of sampled animals across categories of age, history of treatment and BC score is presented in Table 1. Of 384 examined camels, 212 (55.21%) were infested by ticks. Of the 384 camels examined for ticks, 154 (40.1%) were dewormed for external parasites, whereas 230 (59.9%) were non‐dewormed. A higher proportion of adult animals (48.9%) and those with a medium BC (49.5%) were sampled as shown Table 1 and Figure 2.

**TABLE 1:** Distribution of sampled camels across predictor variable categories in the study area.

Risk factors	Categories	Frequency	Percentage
Age	Young	84	21.9
	Adult	188	48.9
	Old	112	29.2
Treatment	Dewormed	154	40.1
	Non dewormed	230	59.9
Body conditions	Poor	64	16.7
	Medium	190	49.5
	Good	130	33.9

### 3.2 Prevalence of Hard Tick Infestation in Camels

Out of 384 examined camels, 212 (55.21%) were infested by ticks. From a total of 1008 ixodid ticks collected, 4 Ixodidae tick genera and 7 species were identified: *Amblyomma variegatum, Amblyomma lepidium, Hyalomma dromedarius, Hyalomma marginatum rufipes, Hyalomma truncatum, Rhipicephalus (Bo.) decoloratus* and *Rhipicephalus pulchelis*.

### 3.3 Tick Genera With Their Predilection Site

**FIGURE 3:**
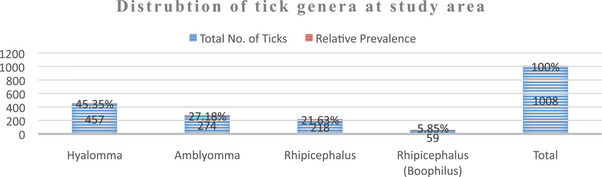
Distribution of tick genera at the study area.

The identified genera of ticks in this study consist of *Hyalomma* (45.354%), followed by *Amblyomma* (27.18%), *Rhipicephalus* (21.63%) and *Rhipicephalus* (*Boophilus*) (5.85%). Additionally, seven tick species belonging to four genera were identified in the sampled animals as shown in Table 2 and Figure 3.

**TABLE 2:** Descriptive information on the tick genera in Habru district, Ethiopia, 2024.

Tick genera	Total no. of ticks	Relative prevalence (%)
*Hyalomma*	457	45.35
*Amblyomma*	274	27.18
*Rhipicephalus*	218	21.63
*Rhipicephalus* (*Boophilus*)	59	5.85
Total	1008	100

### 3.4 Distribution of Tick Species Based on Their Sex in Sampled Animals

**FIGURE 4:**
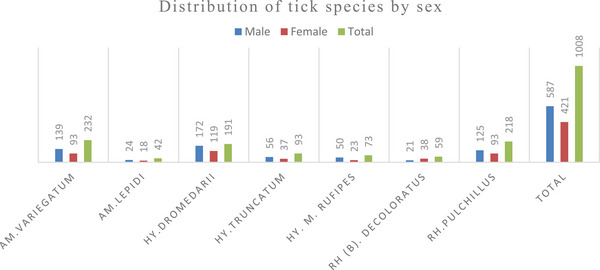
Bar graphical distribution of tick species within sex.

The distribution of tick species: Among the male groups, *A. variegatum, H. dromedarii* and *Rhipicephalus pulchillus* dominate the majority compared to other tick species. In contrast, the female tick species reveal that *H. dromedarii* showed the highest proportion compared to other female tick species. When considering the prevalence of tick species across all sexes, *H. dromedarii* (28.3%), *A. variegatum* (23.02%) and *R. pulchillus* (21.63%) exhibit the highest prevalence compared to the rest of the tick species as indicated in Table 3 and Figure 4.

**TABLE 3:** Distribution of tick species based on their sex and prevalence in the study area.

Tick species	Male	Female	Total	F:M ratio	Prevalence (%)
*A. variegatum*	139	93	232	1:1.49	23.02
*A. lepidium*	24	18	42	1:1.3	4.2
*H. dromedarii*	172	119	191	1:1.3	28.87
*H. truncatum*	56	37	93	1:1.5	9.23
*H. m. rufipes*	50	23	73	1:2.17	7.24
*R. (B). decoloratus*	21	38	59	1:0.55	5.85
*R. pulchillus*	125	93	218	1:1.3	21.63
Total	587	421	1008	1:1.39	100

### 3.5 Distribution of Tick Species Based on Their Predilection Sites

**FIGURE 5:**
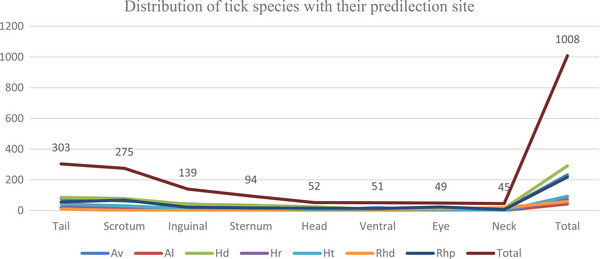
Graphic distribution of tick species based on their predilection site in the study area.

The distribution of tick species across their preferred attachment sites: The tail was the most favoured site, accounting for 30.06%, followed by the scrotum at 27.28%, the inguinal region at 13.79%, the sternum at 9.33%, the head at 5.16%, the ventral area at 5.06%, the eye at 4.86% and the neck at 4.46% as shown blew Table 4 and Figure 5.

**TABLE 4:** Distribution of tick species in different attachment sites of camels in the study area.

Site	*Av*	*Al*	*Hd*	*Hr*	*Ht*	*Rhd*	*Rhp*	Total	Prevalence (%)
Tail	77	12	86	21	42	9	56	303	30.06
Scrotum	62	13	78	25	30	0	67	275	27.28
Inguinal	43	12	41	10	12	0	21	139	13.79
Sternum	25	5	35	8	4	0	17	94	9.33
Head	0	0	24	0	0	13	15	52	5.16
Ventral	19	0	7	0	5	7	13	51	5.06
Eye	0	0	6	9	0	11	23	49	4.86
Neck	6	0	14	0	0	19	6	45	4.46
Total	232	42	291	73	93	59	218	1008	100

Abbreviations: *Al*, *Amblyomma lepidium*; *Av*, *Amblyomma variegatum*; *Hd*, *Hyalomma dromedarii*; *Hr*, *Hyalomma rufipes*; *Ht*, *Hyalomma truncatum*; *Rhd*, *Rhipicephalus (Boophilus) decoloratus*; *Rhp*, *Rhipicephalus (Boophilus) pulchellus*.

### 3.6 Risk Factors Associated With Tick Prevalence

Table 5 displays the findings of the univariable logistic regression model. The putative risk factors such as age group, treatment history and BC score of animals are assessed for their association with the status of tick infestation through univariable risk factor analysis.

**TABLE 5:** Univariable analysis of risk factors with tick infestation status in the study area.

Variables	Categories	*n*	*N*	*p* value	COR (95% CI [LB‐UB])
Age	Young	84	35 (41.67%)	0.151 0.067	1.528 (0.8568521–2.726987) 2.061 (1.087865–3.908155)
	Adult	188	115 (61.17%)		1
	Old	112	62 (55.35%)		
BCS	Good	130	55 (42.3%)		1
	Medium	190	114 (60%)	0.000	3.033 (1.797584–5.118952)
	Poor	64	43 (67.19%)	0.000	3.688 (1.85177–7.346851)
Treatment	Dewormed	154	50 (32.46%)		1
	Not	230	162 (70.43%)	0.00	0.157 (0.0955312–0.257007)

Abbreviations: BCS, body condition scores; CI, confidence interval; COR, crude odds ratio; *N*, proportion of infested animals; *n*, numbers of examined animals.

The multivariable logistic regression analysis showed that poor (AOR: 3.688) and medium (AOR: 3.033) BC scores were 3 times more exposed to tick infestation than good BC scores. The non‐dewormed camels were also 0.16 times more exposed than the dewormed ones as shown in Table 6.

**TABLE 6:** Multivariable analysis of risk factors with tick infestation status in the study area.

Variables	Categories	AOR: 95% CI (LB‐UB)	*p* value
Age	Young	1.529: 0.8568521–2.726987	0.151
	Adult	2.062: 1.087865–3.908155	0.067
	Old	1	
BCS	Good		
	Medium	3.033: 1.797584–5.118952	0.000
	Poor	3.688: 1.85177–7.346851	0.000
Treatment	Deworm	1	
	Non‐deworm	0.157: 0.0955312–0.257007	0.000

Abbreviations: AOR, adjusted odds ratio; BCS, body condition scores; CI, confidence interval; *N*, proportion of infested animals; *n*, numbers of examined animals.

## 4 Discussions

The overall prevalence of tick infestation in the study area was 55.21%. This result closely matched the findings of previous studies: 58.3% in Dire Dawa (Eyeruselam 2008), 61.46% in Eastern Ethiopia (Dinka et al. 2010), 55.5% in Pakistan (Qamar et al. 2018), 59.25% in Iran (Moshaverinia and Moghaddas 2015), 65.77% in Iraq (Shubber et al. 2014) and 68.2% in Saudi Arabia (Alanazi et al. 2020). Nevertheless, it was lower than the reported rates of 97.7% in Borana (Megersa et al. 2012), 97.44% in Yabello (Elias et al. 2020), 96.6% in the Southern Zone of Tigray (Kiros et al. 2014), 94% in Dire Dawa (Taddese and Mustefa 2013), 86.8% in the Somali regional state (Jama et al. 2023), 81.7% in Hararghe (Ahmed et al. 2024), 82.8% in Jigjiga (Hussen and Agonafir 2018), 78.6% in Eastern Ethiopia (Feyera Dewo et al. 2017) and 77.4% in Afar (Feki et al. 2021) within Ethiopia, as well as 98.43% in Iraq (AH et al. 2020), 97% in Somalia (Farah Isse and Ali 2017) and 100% in Egypt (Ghoneim et al. 2020) globally. Furthermore, it was higher than the reported prevalence of 35.6% in Nigeria (Abdullahi et al. 2018). These variations might be associated with different topography of the area, the difference in sample size, the difference in season of examination, the presence or absence of seasonal deworming, temperature difference, different humidity, level of immunity of the animal, amount of rainfall that the areas are receiving, nutritional status of the animal, different management system in various areas and awareness of farmers about parasitic infection. The high prevalence of tick infestation in the study areas is attributed to drought conditions, bimodal rainfall patterns, dense vegetation, lowland regions and the hot and humid climate, all of which create favourable conditions for tick proliferation (SARC [Sirinka Agriculture Research Center] 2015; Alemu et al. 2024).The geography and climate of the area, influenced by factors such as temperature, rainfall, humidity, vegetation, landscape and altitude, have been associated with a higher abundance of ticks. The role of these factors in promoting tick proliferation has been reported in various regions across the country and around the globe (Elias et al. 2020).

There was a statistically significant association (*p value <*0.05) between tick infestation rate and BC of the camels. The poor BC score of camels (67.19%) was more infested than medium (60%) and good BC scores of camels (42.3%). This finding was supported by earlier studies from Ethiopia (Hussen and Agonafir 2018; Feki et al. 2021; Feyera Dewo et al. 2017; Jama et al. 2023; Megersa et al. 2012; Ahmed et al. 2024). Nevertheless, some previous studies stated that BC scores have no significant correlation with the occurrences of tick infestation in camels from various parts of Ethiopia (Elias et al. 2020; Taddese and Mustefa 2013). The higher prevalence in poor body‐conditioned animals might be because poorly conditioned animals were the least resistant to tick infestation and lacked enough body potential to build resistance, whereas over‐conditioned animals showed reasonable combat to the infestation according to Manan et al. (2007). Alternatively, tick infestation might be a cause for poor BC due to the consumption of high amounts of blood by those ticks; hence, high prevalence was computed in this group of animals and may be due to poor BCs. Animals have ruffled hair coats that allow ticks to penetrate hair and attach to the skin of animal easily (Feki et al. 2021; Jama et al. 2023). The prevalence of tick infestation was a significant association (*p value* <0.05) between the treatment (deworming) histories of camels. However, there were no previous findings that were done by using this risk factor variable. The number of non‐dewormed animals (70.45%) was higher infested by ticks than that of dewormed (32.46%) camels. This is due to the reduction of tick burden by effective anthelmintic drugs (ivermectin) and acaricides in dewormed camels.

The prevalence of tick infestation had an insignificant correlation with the age of camels in this study. However, adult camels (61.17%) were more infested than old (55.35%) and young aged camels (41.67%). This insignificant correlation finding was supported by the previous findings from Ethiopia (Elias et al. 2020; Hussen and Agonafir 2018; Feyera Dewo et al. 2017; Ahmed et al. 2024). The higher prevalence in adult animals may be due to outdoor management and the long‐distance movement of adult animals to search for feed and water when compared to older and younger animals, especially calves that remain around the homestead and are less exposed than adult camels. Moreover, the higher prevalence of adult camels is due to the higher numbers of adult camels examined in my thesis (Belete and Mekuria 2023).

*Hyalomma* (45.35%) was the most abundant tick genera, followed by *Amblyomma* (27.18%), *Rhipicephalus* (21.63%) and *Rhipicephalus* (*Boophilus*) (5.85%) in this study. This *Amblyomma* finding (27.18%) was supported by the previous reports from Ethiopia (Feki et al. 2021). Studies report that *Rhipicephalus* were the most prevalent tick genera in different parts of Ethiopia (Elias et al. 2020; Hussen and Agonafir 2018; Feyera Dewo et al. 2017; Kiros et al. 2014; Megersa et al. 2012; Ahmed et al. 2024). The high abundance of ticks is attributed to arid and semi‐arid conditions, high temperatures, low humidity and sparse vegetation, which provide an ideal environment. Their prevalence is further influenced by the availability of suitable hosts and their adaptability to harsh climates (Chaibi et al. 2024). The least tick genera of *Boophilus* were consistent with the previous reports of different studies (Elias et al. 2020; Hussen and Agonafir 2018; Feki et al. 2021; Feyera Dewo et al. 2017; Kiros et al. 2014; Megersa et al. 2012; Ahmed et al. 2024). These variations might be attributed to the preference of *Boophilus* ticks for dry regions with higher humidity, vegetation and moderate, humid climatic conditions. Their abundance is further influenced by the timing of the rainy season and their selective targeting of ruminant hosts (Elias et al. 2020; Taddese and Mustefa 2013).

*H. dromedarii* (28.7%) was the most predominant species infesting camel in the study area. This finding was agreed with in previous study reports in different parts of the world (Moshaverinia and Moghaddas 2015; Alanazi et al. 2020; Farah Isse and Ali 2017; Ghoneim et al. 2020; Yasmeen et al. 2023). Nevertheless, *Rhipicephalus pulchelus* was the most abundant species in previous study reports of Ethiopia (Elias et al. 2020; Hussen and Agonafir 2018; Kiros et al. 2014; Jama et al. 2023; Megersa et al. 2012). Again, *Boophilus decoloratus* was the most abundant species in Nigeria (Abdullahi 2018). *H. dromedarii* (28.7%) occurrence has also concurred with the earlier finding of Eyeruselam (2008), Taddese and Mustefa (2013), Dinka et al. (2010), Feyera Dewo et al. (2017), Hussen and Agonafir (2018) and Elias et al. (2020). They reported that 26.8%, 26.85%, 15.36%, 20%, 20.1% and 27.9%, respectively. Conversely, it was higher than the study of Zeleke and Bekele (2004), Bekele (2010), Megersa et al. (2012) and Abdullahi et al. (2018) who reported that 3.87%, 1.2%, 10.8% and 13.2%, respectively, from Ethiopia and 56.8% (Farah Isse and Ali 2017) and 90.7% (Mohsen et al. 2013) from the world. This difference might be due to management, agroecological and geographical differences. Camels are the preferred hosts of *H. dromedarii* (Walker 2003).

*A. variegatum* (23.02%) was the second most abundant tick species in the study area. This finding was consistent with the findings of 21% (Hamza et al. 2019) and 22.9% (Kiros et al. 2014) Conversely, it was greater than the finding of 15.2% in Nigeria (Abdullahi et al. 2018); 6.5% in Eastern Ethiopia (Jama et al. 2023); 6.2% in Jijjiga (Hussen and Agonafir 2018); 4.7% in Borna (Elias et al. 2020); 3.7% in Somali regional states of Ethiopia (Feyera Dewo et al. 2017) and 2.59% in Dire Dawa (Taddese and Mustefa 2013). The ulcer caused by this tick species becomes a favourable site for secondary bacterial infections like *Dermatophilus congolensis*. *A. variegatum* has great economic importance on cattle because it has an association with heartwater (Cowdrosis) (Walker 2003).

*R. pulchelus* (21.63%) was the third most abundant tick species found in the study area. Nevertheless, it was the most primary abundant species in different parts of Ethiopia (Elias et al. 2020; Hussen and Agonafir 2018; Kiros et al. 2014; Jama et al. 2023; Megersa et al. 2012). On the other hands, the proportion of *R. pulchelus* (21.63%) was lower than the previous finding of 37.5% (Hussen and Agonafir 2018); 46.8% (Taddese and Mustefa 2013); 48.9% (Jama et al. 2023); 69.6% (Megersa et al. 2012); 90.6% (Elias et al. 2020) and 92.7% (Kiros et al. 2014). However, this finding was higher than the finding of 5.3% in Somalia (Hamza et al. 2019). Other remaining tick species like *H. truncated* (9.23%) was among the moderately abundant tick species in this study followed by *H. m. rufipes* (7.24%) and *Rh (Bo) decelerates* (5.85%) which were agreed with the previous findings from Ethiopia (Taddese and Mustefa 2013) and in Sudan (El Tigani and Mohammed 2010).

*A. lepidium* was the least abundant thick in the study area. This finding consistent with the previous reports from Ethiopia (Elias et al. 2020; Hussen and Agonafir 2018; Feyera Dewo et al. 2017; Ahmed et al. 2024). It accounts only 4.2% of the total coverage which was agreed with the 4.9 % (Ahmed et al. 2024), 4.6% (Hussen and Agonafir 2018) and 3.4% (Kiros et al. 2014). The little abundance of this species might be associated with availability of suitable hosts since it prefers a cattle or the climatic factor in the study area. This tick transmits the *Cowdria ruminantium*, which causes heartwater and the protozoans *Theileria mutans* and *Theileria velifera* which cause benign bovine theilerioses (Walker 2003). The great variation of tick burden in different countries may be due to application of acaricides and ivermectin, management practice, production system factors, agro‐ecological, geographical difference, seasonal availability and climatic condition in the pastoral areas (Elias et al. 2020; Taddese and Mustefa 2013; Ahmed et al. 2024; Kifle et al. 2021).

Concerning tick's predilection sites, tail (30.06%) was the most preferred site, followed by scrotum (27.28%) and inguinal (13.79%), whereas eye (4.86%) and neck (4.46%) are the least attachment predilection sites for *H. dromedarii, A. variegatum* and *R. pulchillus* ticks in this study. These findings concur with the previous study reports (Elias et al. 2020; Hussen and Agonafir 2018; Ahmed et al. 2024) who reported that tail and scrotum were the most preferred site for ticks, and back or sides of the camel were the least attachment site of ticks in camels. These variations were related to the possibility of penetration by their hypostome; *Rhipicephalus* attach to the thin skin (ear, head, under tail, the margin of the anus), whereas long‐mouthed ticks (*Amblyomma* and *Hyalomma*) can attach at the ticker skin (ventral, sternum and udder) (Taddese and Mustefa 2013). A variety of factors such as host density, interaction between tick species and inaccessibility for grooming determine the attachment site of ticks (Gebre et al. 2001). With regard to male to the female ratio, in all cases, except for *B. decoloratus*, males outnumbered females. These findings agreed with the previous reports from Ethiopia (Elias et al. 2020; Hussen and Agonafir 2018). The difference is attributed to most probably because fully engorged female ticks drop off to the ground to lay eggs, whereas males tend to remain on the host up to several months later to continue feeding and mating with other females on the host before dropping off (Abebaw 2004). Host grooming easily removes semi‐engorged or engorged females as compared to males. The females of *B. decoloratus* outnumbered males in this study, probably due to the small size of the males, which could not be seen, and this might be one of the contributory factors for missing males (Ayana et al. 2013). These findings were supported by earlier study reports from different parts of Ethiopia (Elias et al. 2020; Hussen and Agonafir 2018; Taddese and Mustefa 2013; Ayana et al. 2013).

## 5 Conclusions

The study area exhibited a moderate tick infestation prevalence of 55.21%. Ticks significantly affected camels in poor BC and those not dewormed (*p value* <0.05). Among the identified tick species, *H. dromedarii* (28.7%) was the most prevalent. Ticks were primarily concentrated around the camel's scrotum and beneath the tail. The findings highlight the widespread presence of ticks in the study area, affecting camels with various tick species. This infestation could result in significant economic losses due to disease transmission and reduced health and productivity of camels. Hence, implementing an effective tick control strategy is essential for the study area.

## Author Contributions

**Yidersal Tizazu**: writing original article, conceptualizations of the study, methodology, data collection. **Tsedalu Yirsa**: supervision, validation, methodology, statically analysis. **Abebe Berihun**: supervision, investigation, software. **Asres Zegye**: statistical analysis, software, supervision. All author(s) read and approved the manuscript.

## Ethics Statement

All procedures and animal care adhered to Federations of Animal Sciences Societies (FASS) (2010) guidelines, and the project was approved by the Animal Welfare and Ethical Review Committee of the School of Veterinary Medicine of Woldia University from Ethiopia (Ref. No. DVM/23/250/2023). All appropriate precautions were taken to reduce the pain the animals engaged in this study endured. Notably, there were no known dangers or discomforts related to taking samples from the study animals.

## Consent

In addition, study participants provided oral consent.

## Conflicts of Interest

The authors declare no conflicts of interest.

### Peer Review

The peer review history for this article is available at https://publons.com/publon/10.1002/vms3.70338.

## Data Availability

The data used for analysis is fully available in the manuscript file without restriction.
